# Subacute Thyroiditis: A Rare Cause of Pyrexia of an Unknown Origin

**DOI:** 10.7759/cureus.67806

**Published:** 2024-08-26

**Authors:** Isuru Perera, Hiruni Fernando, KVC Janaka, Dimithri Gamaarachchi, Manojkumar Krishnan

**Affiliations:** 1 Internal Medicine, Sri Jayewardenepura General Hospital, Colombo, LKA

**Keywords:** pyrexia of unknown origin (puo), thyroid function test (tft), management of subacute thyroiditis, imaging in thyroiditis, subacute thyroiditis

## Abstract

Subacute thyroiditis is inflammation of the thyroid gland, classically presenting with neck pain or discomfort and sometimes with associated diffuse tender goiter and overt hyperthyroid symptoms. Only a few rare cases of subacute thyroiditis presenting as pyrexia of unknown origin (PUO) without any of the aforementioned clinical features have been reported in the literature. A 62-year-old male, with a past history of diabetes mellitus, presented with a history of intermittent fever lasting for one month duration. He did not have any significant localizing symptoms, except for a mild headache, and his examination findings were unremarkable as well. Investigations revealed a high erythrocyte sedimentation rate (ESR), high C-reactive protein (CRP) levels, and a deranged thyroid profile, with high free T3 and T4 and suppressed thyroid-stimulating hormone (TSH) levels, suggestive of subacute thyroiditis. The diagnosis was further reinforced by the findings of a supportive ultrasound scan of the neck. The patient was started on steroids, to which he showed a significant clinical and biochemical response. Here, we aim to highlight atypical presentations of subacute thyroiditis and the importance of early consideration of endocrine diseases in the workup of PUO, sometimes even in the absence of suggestive clinical features.

## Introduction

Subacute thyroiditis is transient inflammation of the thyroid gland characterized by its classical presentation of neck pain and tenderness with features of systemic inflammation and occasionally with associated hyperthyroid symptoms [1]. This is thought to occur due to a viral or post-viral inflammatory process where there is follicular damage and subsequent release of thyroid hormones into the circulation. There is a resultant high free T3 and T4 levels with suppressed thyroid-stimulating hormone (TSH) levels, and in some patients, there can be overt clinical features of hyperthyroidism as well. Systemic inflammatory features such as fever with associated increased biochemical inflammatory markers are also shown to be present with subacute thyroiditis [1].

A febrile illness of more than three weeks is generally considered a pyrexia of unknown origin when a diagnosis is not established after at least three outpatient evaluations or three days of in-hospital evaluation [2]. Subacute thyroiditis is generally not considered a cause for pyrexia of unknown origin (PUO) in clinical practice, probably owing to its classical presentation. Here, we present a case of subacute thyroiditis presenting solely as a case of PUO without the typical symptoms.

## Case presentation

A 62-year-old South Asian male presented to the hospital with a complaint of fever lasting for a one-month duration. The fever was intermittent with one to two fever episodes of around 101 F occurring daily. He complained of malaise and loss of appetite with a significant weight loss of 5 kg over one month. The occurrence of fever did not have any correlation with the time of the day, and he denied having any drenching night sweats. There was no history of any localizing respiratory, urinary, or abdominal symptoms. Apart from a mild headache that was not associated with nausea, vomiting, photophobia, or phonophobia, he did not complain of any symptoms that involved the central or peripheral nervous system. He did not complain of any dysphagia, neck pain, or hoarseness of voice. There was no history of recent hospital admissions or history of recurrent infections. He had taken medications for his symptoms twice as an outpatient and has been investigated once, but the records were not available.

He had a travel history of recent travel to India two months before the onset of symptoms. His past medical history included diabetes mellitus for five years duration. About one month prior to the onset of his symptoms, he had experienced an episode of sore throat with associated mild fever, which resolved spontaneously within one week. His past surgical history and allergic history were unremarkable. His routine medications included metformin 1 g bd, gliclazide MR 60 mg bd, sitagliptin 100 mg, and empagliflozin 10 mg. 

On examination, he was febrile, with a temperature of 100 F. He did not have any lymphadenopathy, oral ulcers, alopecia patches, or any other skin rashes, and he was not pale and anicteric. His blood pressure was 120/70, with a pulse rate of 109. He did not have any cardiac murmurs, and there were no peripheral stigmata of infective endocarditis. His abdomen was soft and non-tender with no palpable organomegaly. His respiratory system, central, and peripheral nervous system examinations were unremarkable. There was a mild neck tenderness, but there was no palpable goiter.

**Figure 1:**
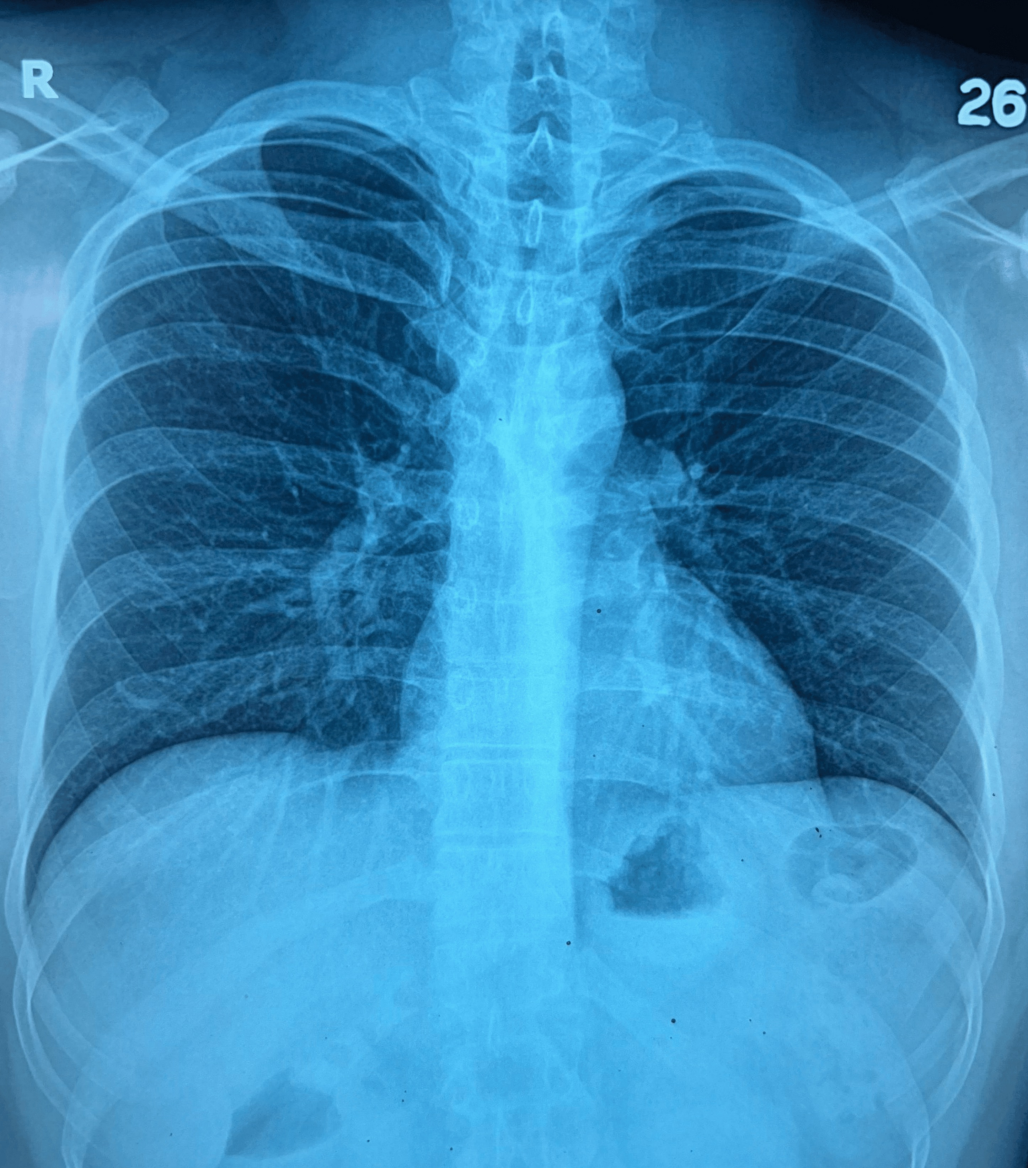
Chest X-ray of a 62-year-old male patient presenting with pyrexia of unknown origin

Routine initial blood investigations were sent, which are summarized in Table 1. Due to the regional prevalence of tuberculosis (TB), screening tests for TB including, three sputum samples for acid-fast bacilli (AFB), Mantoux testing, chest X-ray, and Xpert MTB/RIF assay (Gene Xpert) were sent, all of which yielded negative results (Figure 1). Ultrasound scan (USS) abdomen and 2D echocardiogram were performed, both of which turned out to be negative, the latter specifically negative for any valvular vegetations or intracardiac masses suggestive of infective endocarditis.

**Table 1:** Summary of initial investigations WBC: White blood cells, ESR: Erythrocyte sedimentation rate, CRP: C-reactive protein, LDH: Lactate dehydrogenase, AST: Aspartate transaminase, ALT: Alanine transaminase, ALP: Alkaline phosphatase, PT/INR: Prothrombin time/international normalized ratio

Investigation			Result	Unit	Normal range
Full blood count		WBC	7.7 × 10^3^	µL	4–11 × 10^3^
		Neutrophils	5.87 × 10^3^	µL	2–7 × 10^3^
		Lymphocytes	1.51 × 10^3^	µL	0.8–4 × 10^3^
		Eosinophils	0.02 × 10^3^	µL	0.02–0.5 × 10^3^
		Hemoglobin	13.2	g/dL	11–16
		Platelets	435	µL	150 × 10^3^ to 450 × 10^3^
ESR			60	mm/hour	0–30
CRP			230	mg/L	<6
LDH			250	U/L	200–400
Thyroid profile	TSH		<0.0025	mIU/L	0.4–4.5
	Free T4		2.61	ng/dL	0.7–1.48
	Free T3		4.18	pg/mL	1.58–3.91
Liver enzymes		AST	31	U/L	0–37
		ALT	29	U/L	7–35
		ALP	63	U/L	30–120
Liver functions		Total Bilirubin	1.24	mg/dL	0.2–1.1
		Albumin	4	g/dL	3.4–5.4
		PT/INR	0.9		0.8–1.1
Serum Electrolytes		Sodium	138	mmol/L	136–145
		Potassium	4.8	mmol/L	3.5–5.3
Serum creatinine			88	µmol/L	45–90
Urine full report	Pus cells		1–2	Hpf	
	Red cells		Nil	Hpf	
	Protein		Trace		
Blood picture			Reactive picture with left shift of neutrophils with toxic changes No abnormal cells		
Blood culture			No growth		
Urine culture			No growth		

In view of mild neck tenderness and the elevated free T3 and T4 levels with low TSH, USS neck was planned, which revealed minimally enlarged right and left lobes and isthmus of the thyroid gland, with heterogeneously hypoechoic echotexture, without a significant increase in vascularity, suggestive of thyroiditis. Thyroid peroxidase antibodies were negative.

Initially, the patient was started on IV ceftriaxone 2 g daily prophylactically, and it was continued for five days up until the diagnosis of subacute thyroiditis was established, and the possibility of concurrent bacterial infection was reasonably excluded with the result of normal procalcitonin levels. Once the diagnosis of subacute thyroiditis was established, the patient was started on oral prednisolone 40 mg daily to which he showed a significant clinical and biochemical response. With the continuation of treatment, his CRP levels dropped from 230 mg/L to 90 mg/L and then to 45 mg/L, and he showed a significant clinical response with settling of fever and improved appetite and general well-being.

The patient was discharged on oral prednisolone 40 mg daily and was reviewed in one week. Since he was asymptomatic with normalization of inflammatory markers, his prednisolone dose was gradually tapered off and was omitted after one month of treatment. He was asymptomatic throughout the period after discharge and was reviewed with thyroid profile one month and three months after discharge and those were within normal limits.

## Discussion

Initially, the definition of PUO was a fever of more than 38.3 °C on several occasions lasting for more than three weeks, without being able to arrive at a diagnosis after one week of in-hospital evaluation. This definition was amended in 1991, where four classes of PUO were introduced, namely, classical PUO, nosocomial PUO, neutropenic PUO, and HIV-associated PUO, and the evaluation period was changed to a minimum of three outpatient visits or three days of in-hospital evaluation [2].

Thyroiditis can be broadly categorized into acute suppurative thyroiditis, which is usually due to acute bacterial infections, subacute thyroiditis, and chronic thyroiditis, which commonly occurs in relation to autoimmune pathologies [3]. Subacute thyroiditis can be either subacute granulomatous thyroiditis (de Quervain thyroiditis), which is painful and painless subacute lymphocytic thyroiditis [4]. In general terms, when it is mentioned as subacute thyroiditis in the literature, it is usually referred to as subacute granulomatous thyroiditis [1].

Subacute thyroiditis is thought to occur in relation to viral or post-viral inflammatory processes. When there is follicular damage and disruption of the follicular epithelium due to the inflammatory process, unregulated release of thyroid hormones into the circulation occurs, resulting in overt clinical or biochemical hyperthyroidism. As thyroid hormone stores are depleted over time, the patient can become hypothyroid because new hormone synthesis is also impaired due to the follicular destruction and also due to suppressed TSH levels occurring as a result of the previous hyperthyroid status. In the majority of patients, over time, the thyroid follicles will regenerate and resume hormone synthesis, and the patient will become euthyroid again [1].

Subacute thyroiditis generally presents with pain in the neck with associated fever and sometimes features of thyrotoxicity. Rarely, it can present without any overt features of hyperthyroidism and can present as a fever of unknown origin [5]. ESR and CRP levels can be increased in these cases, which was also observed in our patient [5]. The key to identifying subacute thyroiditis is performing a thyroid profile comprising of TSH, free T4, and free T3 levels where the hyperthyroid status can be biochemically observed with suppressed TSH levels and increased free T3 and T4 levels. Even though the thyroid function tests are deranged in almost all cases of subacute thyroiditis, there have been instances of subacute thyroiditis with normal thyroid function tests with the diagnosis made solely through advanced imaging modalities such as computed tomography (CT) and proton emission tomography (PET) scans [6].

Even though endocrine causes are considered in the etiological assessment of PUO, they are generally not among the first differential diagnoses, especially subacute thyroiditis. This may probably be due to the classical presentation of subacute thyroiditis where the patient would present with neck pain and tenderness with associated systemic inflammatory features and hyperthyroid symptoms. This initial classical presentation will itself be suggestive of subacute thyroiditis and will prevent it from being classified as a case of PUO. However, considering subacute thyroiditis as a differential diagnosis in the early workup of PUO even in the absence of the aforementioned features will prevent the patient from being subjected to excessive unnecessary investigations.

Even though not essential for the diagnosis, it can be reinforced by performing an ultrasound scan of the neck where hypoechoic areas in the thyroid gland can be appreciated. Follow-up ultrasound scans have been shown to be useful in guiding medical therapy where an increase in the number of hypoechoic areas suggests the requirement of a prolonged course of medical therapy [7].

The mainstay in the management of subacute thyroiditis is symptomatic management and alleviating hyperthyroid symptoms if present. If neck discomfort is not very severe and systemic symptoms are not very marked, a trial of only nonsteroidal anti-inflammatory drugs (NSAIDs) or acetylsalicylic acid (aspirin) can be tried. However, if the patient does not respond to it within two to three days or if the patient is having significant neck pain with marked systemic symptoms, treatment with steroids, usually prednisolone 40 mg daily, is recommended, and the duration of prednisolone therapy is guided by the response to treatment [1].

If symptomatic hyperthyroidism is present, to alleviate the symptoms, beta-blockers can be used. It is not recommended to start thionamides to manage hyperthyroidism because thionamides act by inhibiting hormone synthesis and the pathophysiology of hyperthyroidism in subacute thyroiditis is due to excess release of already produced hormones and not due to an increment in hormone synthesis [1].

## Conclusions

Endocrine conditions are an important etiological entity in the workup of pyrexia of unknown origin. Subacute thyroiditis can present as pyrexia of unknown origin even in the absence of local symptoms and symptoms of hyperthyroidism. Hence, including thyroid function tests in the initial workup of PUO cases can aid in the diagnosis of subacute thyroiditis and will prevent the patient from being subjected to unnecessary and extensive investigations. Diagnosis can be further solidified with the use of an ultrasound scan. Management of subacute thyroiditis mainly consists of pain and systemic symptom management with NSAIDs or steroids and management of hyperthyroid features if present.
